# Longitudinal patterns of poverty and health in early childhood: exploring the influence of concurrent, previous, and cumulative poverty on child health outcomes

**DOI:** 10.1186/1471-2431-12-141

**Published:** 2012-09-04

**Authors:** Nikiéma Béatrice, Gauvin Lise, Zunzunegui Maria Victoria, Séguin Louise

**Affiliations:** 1 Department of Social and Preventive Medicine, Université de Montreal, C.P. 6128 succ Centre-Ville, Montreal, Quebec H3C 3 J7, Canada

## Abstract

**Background:**

Although the links between poverty and health have often been studied , the dynamics of poverty and physical health in early childhood remain under-investigated. In particular, it is not known whether the health of young children is affected differently from that of adults by patterns of poverty unique to them.

**Methods:**

We examined patterns of health from 5 to 41 months of age as a function of concurrent, lagged, and chronic exposure to insufficient income. Using data from the first four rounds of the Quebec Longitudinal Study of Child Development, we performed multilevel logistic and multilevel Poisson regressions and latent growth curve analyses to explore associations between exposure to poverty and mother-reported asthma-like attacks, and maternal perception of health status controlling for neonatal, maternal, and environmental characteristics.

**Results:**

The mean number of mother-reported asthma-like attacks significantly decreased as children aged. The likelihood of being perceived in a poorer health status also decreased across time. *Concurrent poverty* was associated with more mother-reported asthma-like attacks and with a higher risk of being perceived in poorer health status. *One-period-lagged poverty* was associated with more mother-reported asthma-like attacks and this remained significant after controlling for concurrent poverty. The number of mother-reported asthma-like attacks was significantly higher among children in the *chronic poverty* class compared to those in the never-poor class, particularly at 17 and 29 months. Perceived health status at 5-months was significantly poorer among chronically poor children compared to never-poor children.

**Conclusion:**

Exposure to poverty negatively affects two major health indicators in early childhood – maternal perception of child health and mother-reported asthma-like attacks. Patterns of the effects vary according to timing and duration of poverty exposure. Further longitudinal research is warranted to disentangle time-specific from cumulative effects of poverty on child health.

## Background

Although the links between poverty and health have often been studied, the dynamics of poverty across time and their association with health outcomes have not been examined extensively among young children. Poverty is not a static condition. There are many transitions in and out of poverty, even more so for parents of young children [1]. Previous research [2-6] suggests that chronic or sustained poverty is more deleterious than intermittent poverty or episodes of shorter duration. However, it is not known whether the health of young children is affected differently from that of adults by patterns of poverty unique to them. Although the importance of poverty related to children’s health during the first years of life for adult health is well established [7-14], there is a dearth of information regarding the dynamics of poverty and health during the early childhood years. Therefore, the main objective of this article was to examine how the timing and duration of episodes of poverty (as measured by income sufficiency) are related to the probability of the mother reporting asthma-like attacks and of perceiving her child to be in less than very good health during early childhood. To this end, we analyzed data from 1998 to 2001 in the Quebec Longitudinal Study of Child Development (QLSCD) birth cohort.

### Existing evidence

Few studies have examined the interrelationships between children’s exposure to poverty at various times in their lives and their health status. Among American children, McLeod and Shanahan [15] and Macmillan et al. [16] found that early childhood exposure to poverty fosters antisocial behaviour and depression later on in life, for example in adolescence. They also found that stability or transitions in parents’ poverty status resulted in changes in children’s antisocial behaviour. MacMillan et al. [16] reported that compared to children who were better-off over the entire study period, those exposed to persistent poverty presented aggravated antisocial behaviour. Longer-term exposure to poverty was associated with increasing antisocial behaviour whereas movement out of poverty did not significantly change trajectories of antisocial behaviour. Strohchein (17) observed somewhat similar patterns in Canada after analysing data on children who were monitored between the ages of 4 and 14 years. In addition to an association between lower household income and higher depression and antisocial behaviour at baseline, the author observed that improvements in income attenuated mental health problems while a decrease in income was associated with an increase in mental health problems. Despite these findings, it should be noted that associations between patterns of poverty and children’s mental health may not replicate across health outcomes. This was exemplified in Strohchein’s study [17]. The association between initial household income and the rate of change in depression became weaker over time whereas the association between initial household income and the rate of change in antisocial behaviour became stronger. In a study conducted by McLeod and Shanahan [15], changes in antisocial behaviour were positively correlated with the number of years spent living in poverty whereas histories of poverty subsequent to baseline status were unrelated to children’s trajectories of depression.

However, compared to mental health, the ways in which children’s physical health are influenced by their household income histories have been less explored. When examining what differentiates early onset from late onset of persistent wheezing in a cohort of children followed from birth to 10 years of age, research has shown that, in addition to a strong genetic component of persistent asthma common to both types, lower social class at birth was related to early onset of wheezing in infancy [18,19]. This effect was not associated with exposure to smoking and to formula feeding which also play a role in respiratory health problems. Another longitudinal investigation involving children from the ages of 6 through 14 years showed that exposure to chronic poverty was associated with the incidence of persistent asthma [20]. Finally another study showed that persistent maternal distress was also associated with a greater likelihood of childhood asthma [21].

Moreover, Chen et al. [3] analyzed how trajectories of socioeconomic status over time were linked to children’s health at 10–11 years and 14–15 years of age. The cumulative effect of low family income over time was more deleterious to children’s physical health than variability in socioeconomic status (measured by the standard deviation in income) or changes in socioeconomic status (measured by income levels). Their results also suggested that exposure to economic hardship before five years of age strongly increases the likelihood of experiencing poor health in later childhood. Other studies have shown that exposure to low income in early childhood is a strong predictor of systolic blood pressure in adolescence, but childhood socioeconomic status seemed unrelated to heart rate or body mass index in the teenage years [22].

### Plausible hypotheses

Four main hypotheses exist regarding the pathways through which exposure to unfavourable socioeconomic circumstances across one’s lifecourse might lead to unfavourable health outcomes. The first hypothesis posits that current exposure to financial difficulties may have an “immediate” effect on the self-reported rating of health status [23,24] or in some cases on mortality [25,26]. A second hypothesis suggests that exposure to deprivation during a particularly sensitive period of development might result in long-lasting and even irreversible damage that will express itself in various forms later on. Studies revealing that early childhood poverty is a predictor of health outcomes in late childhood [3], adolescence [22], or adulthood [25,27] provide some support for this proposition. The third hypothesis focuses on the effects of repeated and sustained exposure to poverty over time. It suggests that individuals exposed to longer durations of deprivation would be at greater risk of poor health. In the Whitehall II study in particular, there was a statistically significant linear relationship between cumulative exposure to poverty over three time-points in the lifecourse and coronary heart disease as well as poor physical and mental functioning [28]. The hypothesis of cumulative poverty has been the one most frequently studied [2-6,29]. Finally, the fourth hypothesis pertains to social mobility. It suggests that fluctuations in household financial status may affect health through social processes associated with upward or downward mobility. For example, Matthews et al. [30] found that downward mobility was associated with a higher incidence of hypertension. Moreover, McDonough et al. [24] suggest that all of these potential pathways may exert an influence and thus each may contribute to explaining a different facet of the poverty-health relationship. In particular, they analyzed the effects of four long-term poverty patterns comprising stable absence of poverty, exiting poverty, entering poverty, and stable poverty, and concluded that different trajectories of poverty affect self-rated health in “distinct ways…in concert with age, education, and race to create gaps in initial health status that were constant over time.”

Framed within this lifecourse perspective, we examined how concurrent poverty, prior poverty occurring at specific times, and cumulative poverty across time may lead to differential patterns of health in a birth cohort of children during the first four years of life. Since the dataset did not allow for a thorough verification of all of the aforementioned hypotheses, the scope of this article was limited to exploring associations between patterns and dynamics of poverty and children’s health across time. We defined concurrent poverty as that level of poverty which was present when the child’s health status or condition was measured. Poverty measured at a previous round or one-period lagged allowed us to explore whether or not poverty at this specific, recent critical period might have subsequent effects on health beyond current poverty. Cumulative poverty designated a child’s membership in chronic or transient poverty classes over the four survey rounds under investigation and allowed for an examination of the impact of repeated and sustained poverty on health.

## Methods

### Data

We used data from the Québec Longitudinal Study of Child Development (QLSCD), an ongoing population-based birth cohort study coordinated by the *Institut de la Statistique du Québec* (ISQ) (Québec Institute of Statistics). The QLSCD started with a representative sample of 2 120 singleton infants who were recorded in the registry of Quebec births in 1997/1998. Excluded were children born to mothers residing in Cree and Inuit territories, Indian reserves, and/or certain northern regions of Québec (2.1% of singleton live births). Infants born before the 24^th^ or after the 42^nd^ week of gestation (0.1%) and those with unknown gestational age (1.3%) were also excluded. Children were enrolled at a mean age of five months (15–36 weeks) and subsequently followed-up annually. We used data collected during the annual rounds between 1998 and 2001 (ages approximately 5, 17, 29, and 41 months) including repeated assessments of children’s health, growth, and use of health care, parents’ health and socioeconomic status, and various characteristics of the child-rearing environment. Much of these data are based on mothers’ responses to computer-assisted, face-to-face interviews administered by trained interviewers, and to self-administered paper questionnaires. We obtained information on the children’s neonatal health status from medical records.

The study received ethical approval from the Research Ethics committee of the Faculty of Medicine , University of Montreal and from the Ethics committee of the Direction Santé Québec, Institut de la statistique du Québec. Parents of participating children gave their signed consent at each study round.

### Child health assessment

For each survey round under study, we classified children according to whether their mothers perceived their child’s health status as being *less than very good* (Yes/No) during the previous 12 months. We then calculated *the number of asthma-like attacks* the mother reported her child had experienced during the same period. As other authors have reported [31-33] it is difficult to accurately diagnose asthma before the age of 6 years as other conditions such as viral infections and bronchitis can be responsible for asthma-like symptoms in young children and toddlers. When children get older, they can be said to have experienced a transient wheeze (which goes away) or to have a persistent wheeze (which is eventually diagnosed as asthma). Although in the QLSCD mothers were asked to report on the number of asthma attacks, we elected to label this outcome as the number of mother-reported asthma-like attacks because these reports encompass both transient and persistent wheezing (whether they are related to asthma or other conditions). Maternal perception of her child’s health status was measured at 5, 17, 29, and 41 months whereas the number of mother-reported asthma-like attacks was measured at 17, 29, and 41 months.

### Operationalization of poverty

At each study round, mothers were asked to report total annual household income before taxes. The interviewer first asked whether or not the household had received any income from a list of possible sources during the preceding 12 months. Respondents were then asked to give their “best estimate of the total income before taxes and deductions of all household members from all sources” [34] (p. 12–16) by indicating the income group in which their total household income fell.

During each round, poverty status was established as a function of living in a household with an insufficient annual income. The threshold for insufficient income was defined as having a total reported income in the previous 12 months below that of the Canadian low-income cut-off (LICO) computed annually by Statistics Canada [35]. LICOs are based on the size of the households and the geographic location of communities in which families reside. For example, in 2001 (4^th^ round of the QLSCD), LICOs were $24 502, $28 101, and $35 455 (CAD) for a family of four living in rural areas, towns (< 30,000 inhabitants), or large cities (> 500,000 inhabitants) respectively [36].

### Other predictors of child health

In addition to poverty, other potential predictors of children’s health included their socio-demographic characteristics (birth order and sex), neonatal health condition (premature status and presence of malformations), and environment and rearing conditions (duration of breastfeeding, having a pet at home, exposure to smoking at home, type of child care, and type of family). Mother-related predictors included the mother’s age, education, and immigrant status. The variables, type of family, type of child care, and exposure to smoking, were observed at each survey round. All other predictors were baseline measures.

#### Statistical analysis strategy

After computing descriptive statistics, we applied multilevel analysis techniques to assess univariate and multivariable associations between various static and dynamic operationalizations of poverty and child health. This approach was used to address repeated measures of dependent outcomes and the mix of time-changing and time-invariant covariates. Multilevel models for count data (Poisson) were fit for associations between poverty and the number of mother-reported asthma-like attacks and multilevel models for binary data (logistic) were fit for associations between poverty and being perceived in less than very good health.

To assess concurrent effects of poverty and those of poverty during the previous survey round, we proceeded with multilevel growth curve analysis in which two hierarchical levels were defined: study rounds (level 1) were nested within children (level 2). Growth curve analysis provided us with a means of describing patterns of change (from baseline status) in the two health indicators as the children aged, and of verifying whether or not both the baseline health status and subsequent change rates varied with the presence of poverty, while controlling for other covariates. Given that at baseline, 75% of children were 5-months old, 8% were 4-months old, and 17% were older than 5 months (6-months = 16%; 7-months = 1%), we believe that inclusion of dummy variables accounting for time coterminously accounted for age.

Reported income, from which we established poverty status, is known to be often subject to measurement errors which may compromise the accuracy of single- or multi-year assessments of poverty [37]. We accounted for this potential error through instrumental variable estimation using the 2-Stage Least Square approach as recommended in the literature [38-40]. In the first stage, annual household income was expressed as a ratio of the relevant LICO. Then, we conducted multilevel linear growth curve analysis in the resulting natural logarithm of the “LICO-standardized” annual household income. For each survey round, the predictors were the household income rank in the sample, the type of family, the mother’s education and immigrant status at baseline, and the child’s birth order. Household income rank has been used in other studies as a valid instrument for measured income [41,42] and was considered an appropriate instrumental variable in our study because it is correlated with income and uncorrelated with the error term in the poverty-health relationship [38]. Only children who participated in all four survey rounds under study were included in these analyses.

In the second stage, the fitted income values were used to establish a child’s poverty status, defined as the exponential of its fitted annual income below the LICO. The measure of concurrent poverty shared the same 12-month-recall period as the health indicator, whereas poverty at a previous survey round referred to poverty status during the 12 months preceding the measurement of the health indicator. For example, at 41 months, concurrent poverty referred to this status between the ages of 29 and 41 months whereas previous poverty referred to this status between the ages of 17 and 28 months. In addition, dummy variables were created for each survey round and were introduced in the first model to capture changes in outcome variables across time. The baseline parameter was set to represent the intercept for the outcome and the other time-specific parameters set to represent change across time. Adjusted poverty variables were added in the second model. Then other predictors were added one by one to assess their confounding role in the poverty-health relationship. They were considered confounders if their inclusion changed the value of the poverty coefficient by at least 10% at any survey round. The final model was fitted with adjusted poverty variables, outcome-specific confounders, and other covariates bearing a statistically significant association with the health outcome.

To assess cumulative effects due to repeated exposure to poverty, we conducted latent class growth modelling to identify longitudinal patterns of poverty over the four survey rounds under study, using the procedure recommended by Muthén and Muthén [43] (p. 185–220). We ran models including 2, 3, and 4 classes of poverty, both linear and quadratic trends, and then compared the Bayesian Information Criterion (BIC) across models. The best fitting model and the one that provided the most reasonable and parsimonious definition was the 3 class model. Hence, children were categorized according to their most likely class membership into one of three poverty classes – those who were never poor, those having experienced transient poverty, and those with chronic poverty. We then used the children’s class membership as a second-level (child-level) variable in a regular growth curve analysis to assess whether or not health at different times varied across poverty latent classes.

For all analyses, sample weights provided by the *Institut de la Statistique du Québec* were used to adjust for non-response bias occurring through attrition. HLM software (v. 6.04) was used to perform logistic and Poisson growth curve analyses and Mplus software (v. 5.2) was used for Latent Class Growth Analysis. For multilevel logistic and multilevel Poisson regressions we applied the sixth order Laplace approximation which provides very accurate estimates for all parameters [44,45]. Details on the analysis strategies and equations of the aforementioned models are presented in Additional file 1: Appendix 1 and Appendix 2 (on-line Additional file 1) respectively. Statistical significance was evaluated at a Alpha level of 1% rather than 5% to provide more conservative estimates.

## Results

### Descriptive

Data were available for 96.5% (N = 2 045), 94.2% (N = 1 997), and 92.0% (N = 1 950) of the original cohort in the 2^nd^, 3^rd^, and 4^th^ annual survey rounds respectively. Table 1 shows the distribution of health indicators and time-dependent covariates at ages 5, 17, 29, and 41 months. The proportion of mothers who perceived their child as being in less than very good health varied between 8% and 11% of cases depending on the survey year. The average number of mother-reported asthma-like attacks per year varied from 0.27 at 17 months to 0.17 at 41 months. Based on reported income levels, the proportion of children who were living in a household with insufficient income varied from 27.6% at 5 months to 20.1% at 41 months. Based on predicted income in the model, the proportion of children living in a household with insufficient income varied from 27.8% at 5 months to 22.4% at 41 months. Table 2 presents the distribution of time-invariant covariates in the sample and as a function of children’s poverty status at baseline. Latent class analysis of poverty across time revealed that 68.8% of children were most likely to be in the never-poor class, 14.7% in the transient poverty class, and 16.5% in the chronic poverty class.

**Table 1:** Child health indicators and time-varying covariates in the initial four rounds of the QLSCD (1998–2001)

	**Survey round**			
**Age of child**	5 months^†^	17 months^†^	29 months^†^	41 months^†^
**Total sample size**	2 120	2 045	1 997	1 950
**Health indicators**				
Number of mother-reported asthma-like attacks* - past 12 months: **M (SD)**	-	0.27 (1.66)	0.23 (1.42)	0.17 (1.11)
Maternal perception of child’s health as being less than very good - past 12 months : **%**	7.9	11.2	9.7	11.0
**Time-varying covariates**				
Household income sufficiency - past 12 months	N=2 078	N=2 007	N=1 968	N=1 893
Sufficient : **%**	72.4	76.0	78.0	79.9
Insufficient : **%**	27.6	24.0	22.0	20.1
Predicted household income sufficiency††	N=1 886	N=1 884	N=1 886	N=1 894
Sufficient : **%**	72.3	74.4	76.7	77.6
Insufficient : **%**	27.7	25.6	23.3	22.4
Type of family - past 12 months	N=2 112	N=2 041	N=1 993	N=1 945
Two biological parents : **%**	80.0	78.0	76.1	75.0
Two parents, one biological : **%**	10.8	11.2	11.2	11.6
Single parent : **%**	9.2	10.8	12.7	13.4
Parents smoke at home *-* past 12 months	N=2 119	N=2 038	N=1 989	N=1 936
No / occasionally : **%**	75.5	69.9	72.9	74.6
Daily : **%**	24.5	30.1	27.1	25.4
Type of child care *-* past 12 months	N=2 120	N=2 045	N=1 997	N=1 950
In child’s home : **%**	90.0	53.6	49.6	36.4
External home-based daycare : **%**	8.3	36.6	33.6	32.7
Daycare centre : **%**	1.7	9.8	16.8	30.9

*Numbers and percentages at 17 months of age pertain to mother-reported asthma-like attacks having occurred since birth.

M (SD): Mean (Standard Deviation); †Weighted percentages.

††Predicted among the 1,950 children who were followed-up to age 41 months.

**Table 2:** Child’s and mother’s characteristics as a function of reported income sufficiency at baseline (5 months) among 1 950 children followed-up to age 41 months (QLSCD, 1998–2001)

	**Distribution in the sample**	**Distribution by reported income level at baseline (5 months)**		
		**Income**		
		**Sufficient**	**Insufficient**	**Missing data**
	**%**	**%**	**%**	**%**
**Child’s birth order**				
First	44.9	45.4	39.9	47.4
Second	39.3	40.5	37.2	36.8
Third and higher	15.8	14.0	22.9	15.8
**Child’s sex**				
Girl	49.8	50.1	45.4	55.3
Boy	50.2	49.9	54.6	44.7
**Mother’s age**				
<20 years	2.9	0.9	8.8	10.5
20-34 years	83.6	85.7	78.1	81.6
>=35 years	13.4	13.3	13.1	7.9
Missing data	0.0	0.1	0.0	0.0
**Mother’s immigrant status**				
Non-immigrant or European-origin immigrant	92.8	96.2	75.7	76.3
Non-European Immigrant	7.1	3.8	24.3	21.1
Missing data	0.1	0.1	0.0	2.6
**Mother’s education**				
College or university studies	64.0	70.4	41.3	44.7
High school, vocational or trade school diploma	20.6	20.1	22.7	34.2
No high school diploma	15.3	9.5	35.8	21.1
Missing data	0.1	0.1	0.2	0.0

### Changes in health status across time

**Figure 1:**
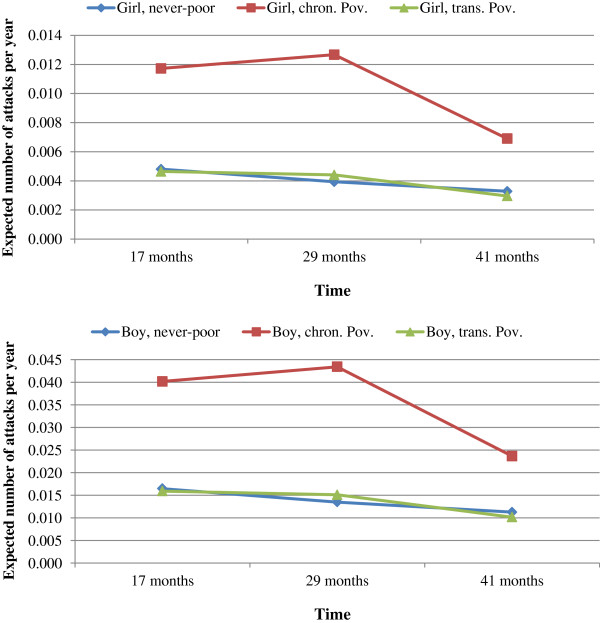
**Expected mean number of mother-reported asthma-like attacks per year, as a function of poverty latent class membership.** Weighted estimates from multilevel Poisson regressions with 1825 children, (QLSCD, 1998-2008).

**Figure 2:**
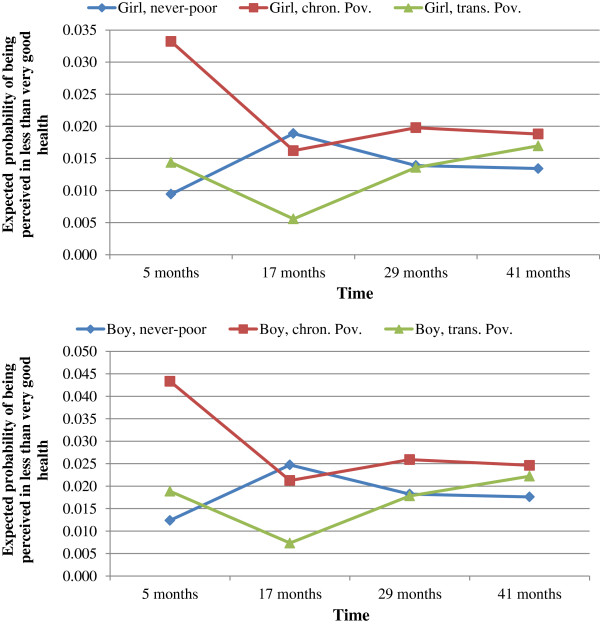
**Expected probabilities of being perceived in less than very good health, as a function of poverty latent class membership.** Weighted estimates from multilevel logistic regressions with 1825 children (QLSCD, 1998-2008).

Adjusted models for the effects of concurrent, previous-period, and cumulative poverty are summarized in Tables 3 and 4 and in Figures 1 and 2. Tables 3 and 4 indicate that the mean number of mother-reported asthma-like attacks decreased as children aged. The likelihood of a mother perceiving her child as being in less than very good health was higher in the second survey round (17 months of age) compared to the initial round (5 months).

**Table 3:** Influence of concurrent poverty on child health in the first 41 months of life, adjusted event rate ratios and odds ratios (ERR & OR) from multilevel Poisson and logistic regressions, (QLSCD, 1998–2001)

	**Number of mother-reported asthma-like attacks during the previous 12 months**^1†^		**Being perceived in less than very good during the past 12 months**^2†^	
	**ERR (95% CI)**		**OR (95% CI)**	
Change from first round (baseline)				
5 months	-		1.00	
17 months	1.00		1.55 (1.18,2.03)^***^	
29 months	0.95 (0.89,1.02)		1.32 (1.01,1.73)^*^	
41 months	0.63 (0.58,0.68) ^***^		1.35 (1.03,1.77)^*^	
Change due to concurrent poverty				
Sufficient income	1.00		1.00	
Insufficient income	1.39 (1.09,1.76)^***^		1.51 (1.14,1.99)^**^	
Number of children	1 944		1 945	
Number of measurements	5 712		7 632	

^1^Adjusted for child’s sex and birth rank, type of family, and mother’s age and education.

^2^Adjusted for child’s sex and birth rank, use of daycare centre, and mother’s education and immigrant status.

^*^p <0.05; ^**^ p <0.01; ^***^ p <0.001.

^†^Weighted parameter estimates.

**Table 4:** Influence of previous-round poverty on child health in the first 41 months of life, adjusted event rate ratios and odds ratios (ERR & OR) from multilevel Poisson and logistic regressions, (QLSCD, 1998–2001)

	**Number of mother-reported asthma-like attacks during the past 12 months**^**1†**^		**Being perceived in less than very good health during the previous 12 months**^**2†**^	
	**ERR (95% CI)**		**OR (95% CI)**	
Change from first round				
17 months	1.00		1.00	
29 months	0.96 (0.90,1.03)		0.84 (0.64,1.09)	
41 months	0.63 (0.58,0.68)^***^		0.85 (0.66,1.10)	
Change due to poverty estimate in the previous round				
Sufficient income	1.00		1.00	
Insufficient income	1.28 (1.08,1.51)^**^		1.28 (0.89,1.84)	
Change due to poverty estimate in the previous round after controlling for current poverty status				
Sufficient income	1.00		1.00	
Insufficient income	1.25 (1.06,1.48)^**^		1.21 (0.82,1.79)	
Number of children with complete data	1 944		1 944	
Number of measurements	5 683		5 687	

^1^Adjusted for child’s sex and birth rank, type of family, and mother’s age and education.

^2^Adjusted for child’s sex and birth rank, use of daycare centre, and mother’s education and immigrant status.

^*^ p < 0.05 ^**^ p < 0.01 ^***^ p < 0.001.

^†^Weighted parameter estimates.

### Effects of concurrent poverty

Over the follow-up periods, after accounting for outcome-specific covariates and applying longitudinal sample weights, concurrent poverty was significantly associated with higher likelihood of being perceived in less than very good health (odds ratio [OR] =1.51; 95% confidence Interval [CI]: 1.14,1.99) and with a higher mean number of mother-reported asthma-like attacks (ERR = 1.39; 95% CI:1.09, 1.76)(Table 3).

### Effects of one-period lagged poverty

Table 4 shows that poverty assessed during the previous survey round had an effect on only one of the indicators. Holding other covariates constant, children exposed to poverty 12 months before the assessment of the health outcome had 28% increase in mother-reported asthma-like attacks (event rate ratio [ERR] = 1.28; 95% CI: 1.08, 1.51) than non-poor children. The increase in the mean number of mother-reported asthma-like attacks remained significantly associated with previous-round poverty (ERR = 1.25; 95% CI: 1.06, 1.48) even after controlling for concurrent poverty.

### Effects of cumulative poverty

Figures 1 and 2 show estimates of the children’s health at different times related to their membership in the three latent classes of poverty and their sex. First-born girls who were classified in the never-poor trajectory group, who were born to 20–34 years old mothers with a university diploma, and who were living in a two-parent-family at 17 months of age represent the reference group. Among this reference-group, the mean number of mother-reported asthma-like attacks significantly decreased across time. In comparison, at 17 months, the number of mother-reported asthma-like attacks per year among children who experienced chronic poverty was 2.44 greater than (from Figure 1, [Event Rate Ratio = 0.012/ 0.005] for girls, and [Event rate Ratio = 0.040/0.016] for boys) that of children who remained on a never-poor trajectory. However, this association was not statistically significant at the 1% alpha level. At 29-months, the burden of mother-reported asthma-like attacks significantly (p < 0.001) increased among chronically poor children (from 0.012 to 0.013 attacks per year among girls, and 0.040 to 0.043 attacks per year among boys) while it decreased among never-poor children (from 0.005 to 0.004 attacks per year among girls, and 0.016 to 0.013 attacks per year among boys), bringing the Event Rate Ratio to 3.22. In both never-poor and chronic poverty groups, the rates of mother-reported asthma-like attacks decreased from ages 29-month to 41 months. Attacks were significantly more frequent among boys compared to girls at baseline with no significant differences in change rates at subsequent follow-ups. There were no significant difference between children belonging to the transient poverty trajectory group and those from non-poor group in terms of both level and change rates for this health indicator.

For the mother’s perception of her child’s health, the reference group included non-poor first-born girls, who used a daycare centre at five months of age, and were born to Canadian-born and University-educated mothers. In Figure 2, perceived health status at five months of age was more likely to be worse (p < 0.001) among chronically poor children (probability =3.3% for girls and 4.3 for boys) compared to non-poor children (probability = 0.9% for girls and 1.2% for boys). The likelihood of being perceived to be in less than very good health status subsequently tended to decrease among children in the chronic poverty class but the association was not statistically significant. Inversely, among children in the non-poor class, the likelihood of being perceived to be in less than very good health status tended to increase with a statistically significantly increase at 17-months of age (probability =1.9% for girls and 2.5% for boys; p < 0.001), followed by non significant associations at 29 and 41 months of age. The differences between children on the transient poverty and those on the never-poor trajectory as well as differences between girls and boys were not statistically significant at the 1% alpha level.

The results for full models with all covariates are available in Additional file 1: Appendix 3 (on-line Additional file 1). They show, *inter alia*, that irrespective of poverty status, living in a single-parent household resulted in an increase in the annual mean number of mother-reported asthma-like attacks. Daycare centre utilization resulted in a greater likelihood of being perceived in less than very good health by the mother.

## Discussion

Using data from a unique birth cohort in Québec, we explored the influence of poverty, its timing, and its accumulation on the number of mother-reported asthma-like attacks and the mother’s perception of her child’s health from birth to 41 months. In summary, results suggest that different dynamics of poverty contribute to the observed negative health outcomes among children living in poverty: *Concurrent poverty* is associated with more mother-reported asthma-like attacks and with a higher risk of being perceived in poorer health status. *One-period-lagged poverty* is associated with more mother-reported asthma-like attacks and this remained consistent after controlling for concurrent poverty. The number of mother-reported asthma-like attacks is significantly higher among children in the *chronic poverty* class compared to those in the never-poor class, particularly at 17 and 29 months. Perceived health status at 5 months was more likely to be less than very good among chronically poor children compared to never-poor children. For both outcomes, the magnitude of the difference between the never poor and the chronically poor children tend to decline over time. Below, we first discuss children’s experience of poverty and their experience of ill-health across time before discussing results on the longitudinal poverty-health relations.

More than one quarter of the children (27.6%) were born into households with insufficient income. The proportion of poor children decreased to about 20% at 41 months of age. At that age, 15% had experienced transient poverty and 17% had spent their entire first 3.5 years of life in a household with insufficient income. Strictly considered, the LICO provides a means of assessing relative rather than absolute poverty. It provides a means of identifying “those who are substantially worse off than the average” [46](p 56). The LICO is currently the most widely accepted measure of deprivation in Canada, and is often cited in research and public policy discussions. The rates of poverty among young children observed here were similar to those for households with children under 18 years of age reported by the Canadian Council on Social Development [47].

The results showed that on average, experience of ill health among QLSCD children varied across time. Taking into account children’s sex and birth order, maternal age, education and immigration status, day-care utilization, single parenthood, and birth order variability, the number of mother-reported asthma-like attacks decreased over time. The decrease in the mean number of mother-reported asthma-like attacks is consistent with the normal history of asthma symptoms in that the majority of symptoms likely develop in the preschool years and decrease later towards remission. Sears [48] reviewed studies suggesting that the highest incidence of asthma occurs during early childhood, followed by a decrease in later childhood. A longitudinal study conducted in Tucson showed that 59.2% of children who developed wheezing before their 3^rd^ birthday were symptom-free when followed up at 6 years of age [31]. Our results suggest that decrease in symptom frequency may be observed at earlier ages.

The likelihood of being perceived in less than very good health by the mother tended to increase across time, but this increase was statistically significant only at 17 months. We are unaware of any study that has longitudinally analyzed how the mother’s perception of her child’s health evolves as the child ages. Studies among adults show that self-rated health declines slowly across time [24,27,49], but we have no reason to expect similar patterns among young children. However, research on inequalities in child health shows that socioeconomic differences in health risks significantly narrow in adolescence, suggesting that we would expect young children’s health to improve as they approach adolescence [50-53].

Turning to the poverty-health relationship, our study confirmed with different analytic strategies the deleterious impact of poverty on aspects of child physical health. In particular, the study showed that living in a household with insufficient income, compared to the absence of poverty, results in more frequent mother-reported asthma-like attacks and a higher likelihood of being perceived in less than very good health. The added value of our study resides in showing that the timing of exposure to poverty potentially affects children’s health in terms of these two health indicators during early childhood.

Contemporaneous poverty predicted worse overall health status as measured by maternal perception of health being less than very good. These results support the hypothesis of “immediate” effects which is suggested in the literature as a possible explanation of the pathways from poverty to ill-health [22,24].

Irrespective of other covariates, insufficient income reported during the previous survey round was significantly associated with the number of mother-reported asthma-like attacks. This influence remained statistically significant even after controlling for concurrent poverty status. As shown in Table 4, the effect of previous-round poverty on the number of mother-reported asthma-like attacks suggests an effect in addition to an immediate effect (i.e. critical period) but could also suggest a cumulative effect of the previous time period. These findings are consistent with those of previous studies which link early childhood poverty to aspects of poor health in adolescence [22] and adulthood [25,27,54], consistent with the hypothesis that poverty can have a delayed or cumulative effect for this outcome.

Evidence of poor health as a function of cumulative exposure to poverty was observed for mother-reported asthma in our study. Membership in the chronic poverty latent class was associated with a higher mean number of mother-reported asthma-like attacks per annum. Although our data may lack statistical power to clearly show differences in mother-reported asthma-like attacks related to poverty in the baseline round despite an OR of 2.44, an increased risk of mother-reported asthma-like attacks from baseline to 29 months was indeed observed among children in the chronically poor group in comparison to a statistically significant decrease in the non-poor group. Previous studies have reported conflicting results on the relationship between poverty and asthma among children [55]. Some studies have reported a higher prevalence of asthma among less privileged children compared to privileged children [56,57] while others have reported no association between poverty and childhood asthma [58,59]. The use of different measures of poverty from one study to another may explain in part this incongruence. Interestingly, the effects of poverty on the severity of asthma attacks seem less controversial. Poverty seems to be associated with more severe cases of asthma attacks [56,57,60]. In our study, chronic exposure to poverty was associated with maternal perception of less than very good child health, but the effect of poverty was evident during the first 17 months of life.

Care was taken to mitigate the effect of measurement errors associated with an income-driven poverty measure on the accuracy of our estimates. Use of instrumental variables is an accepted means of correcting for this type of measurement error. We verified that the household income rank satisfied the main conditions of representing a good instrumental variable for the relationship between poverty and health in childhood. Multilevel procedures for repeated measures accounted for autocorrelation while latent class analysis helped in isolating chronic poverty from transient poverty so that potential residual measurement errors in the former could be captured. However, it is possible that the transient poverty class represents such a heterogeneous group of children that the meaning of any patterns would be difficult to interpret. The lack of a significant association between transient poverty and the two health indicators under study may therefore reflect this measurement shortcoming.

Given this limitation and the fact that we had only three to four measurement occasions depending on the indicator, we were unable to adequately verify the four hypotheses underlying the poverty-health relationship in early childhood. Nevertheless, this limitation does not jeopardize the conclusion that the timing of poverty and chronic poverty matter to children’s physical health in the first few years of life and that the patterns of these effects may differ depending on the outcome. Another limitation of the study is that we did not correct for the QLSCD's design effect, which increases the risk of falsely rejecting the null hypothesis at 5%. However, in keeping with methodological suggestions, we used a conservative alpha level of 1% [61]. At this significance level, the effects of previous round poverty status and cumulative poverty on number of mother-reported asthma-like attacks was still statistically significant as was the association between concurrent poverty and maternal perception of the child’s health status. Finally, the measures are based on parents’ reporting which may include subjectivity and/or memory errors. In particular, one may question the validity of maternal perception of the child’s health. However, we showed in a previous study that when children were 17 months old, the mother’s perception of the child’s health was significantly associated with the presence of acute and chronic health problems and specifically with the occurrence of mother-reported asthma-like attacks [62]. This suggests that maternal perception of the child’s health in the QLSCD is a measure with acceptable validity.

Sample attrition is common in longitudinal studies like the QLSCD. With 92% of the initial sample still participating at the third annual round, the low attrition of the QLSCD is quite exceptional. Nevertheless, children from poor households are over-represented among children lost to follow-up. It is thus possible that we are underestimating the magnitude of poverty effects when detected and we may be unable to detect any effects when in fact they do exist.

## Conclusion

The aforementioned limitations do not offset the strengths of the present contribution to knowledge. Infant and toddler exposure to poverty is associated not only with lower baseline health status but also influences two important indicators of child health during the first four years of life. Indeed, exposure to and timing of poverty appear to be associated with maternal perception of child health as being less than “very good” and an increased risk of asthma attacks in early childhood. In addition, the effects of insufficient income on certain child health indicators seem more marked in the first year and a half of life. Although further longitudinal investigations are required to disentangle time-specific from cumulative effects of exposure to poverty, our findings provide additional impetus to search for effective means to substantially reduce or even eradicate early child poverty in rich countries.

## Abbreviations

LICO: Low-income cut-off; QLSCD: Quebec Longitudinal Study of Child Development.

## Competing interests

The authors declare that they have no competing interests.

## Authors’ contributions

BN analyzed the data and prepared the first draft. LG provided methodological guidance. LG, MVZ, and LS contributed to discussions on analyses and interpretation of results, and revised the manuscript. All authors approved the final version of the manuscript.

## Pre-publication history

The pre-publication history for this paper can be accessed here:

http://www.biomedcentral.com/1471-2431/12/141/prepub

## Supplementary Material

Additional file 1 This additional file is organized in three parts: **Appendix 1.** presents details on the modelling steps as well as a summary of variables tested, excluded, and included by health indicator Equations for baseline and final models are given in **Appendix 2.** The results for full models with all covariates and level 2 variances are presented in **Appendix 3.** Click here for file
