# Supplementary material for: Longitudinal patterns of poverty and health in early childhood: exploring the influence of concurrent, previous, and cumulative poverty on child health outcomes
Source: BMC Pediatr. 2012 Sep 4;12:141. doi: 10.1186/1471-2431-12-141 (PMC3539903; doi:10.1186/1471-2431-12-141)
Supplement: Additional file 1 — This additional file is organized in three parts: Appendix 1. presents details on the modelling steps as well as a summary of variables tested, excluded, and included by health indicator Equations for baseline and final models are given in Appendix 2. The results for full models with all covariates and level 2 variances are presented in Appendix 3. [file 1471-2431-12-141-S1.pdf]

Appendix 1: Models and Tests

A. Model steps

For all models, sample longitudinal weights were applied and EM Laplace-6 estimates were requested.

Steps for the effects of concurrent poverty and previous-period poverty status were as follows:

1- Baseline model with no covariates.
2- Inclusion of binary indicators of survey round to assess time trend for the health indicator. The first time point was not included because it represents the baseline health status (i.e., the reference category).
3- Inclusion of the poverty measure:
   a. poverty status at time "t" for concurrent poverty
   b. poverty status at time "t-1" for previous-period poverty
   c. poverty class membership for cumulative poverty.
4- Inclusion, one by one, of time-varying and time-invariant covariates which were significantly associated ($p \leq 0.05$) with both the poverty and the health indicator at baseline.
5- Full model including the time variables, concurrent / previous-period poverty, and all covariates which remained statistically significant or changed the poverty-coefficient by at least 10%.
6- Exclusion from the final model of non-significant covariates, if their exclusion did not substantially modify the coefficients of the remaining covariates.

B. Summary of variables tested, excluded, and included by health indicator, Quebec (QLSCD 1998-2001).

	Number of mother-reported asthma-like attacks - previous 12 months		Health perceived < very good – previous 12 months	
	Intercept	Slope	Intercept	Slope
**Level 1 variables**				
Time dummy variables				
5 months	-	-	X (ref)	
17 months	X (ref)			X
29 months		X		X
41 months		X		X
Concurrent poverty	Poverty at 17, 29, 41 months		Poverty at 5, 17, 29, 41 months	
Use of daycare centre	✓		Daycare at 5, 17, 29, 41 months	
Living in a single-parent household	Single parenthood at 17, 29, 41 months		✓	
In-home tobacco smoke	✓		✓	
**Level 2 variables**				
Latent classes of poverty	X	X	X	X
Child's birth order			X	
Gender	X		X	
Preterm birth	✓		✓	
Malformation	✓		✓	
Duration of breastfeeding	✓		✓	
Mother’s immigrant status	✓		X	
Mother's education level	X		X	
Mother's age at child’s birth	X		✓	
Having a pet at home at baseline	✓		-	

X : included ; - : Not tested or unmeasured; √: Tested and then excluded from the model

Appendix 2 Equations for baseline and final models

Note: *i* represents repeated measures whereas *j* represents persons

A-Concurrent poverty (Fit with HLM 6.0 software)

A.1-Number of mother-reported asthma-like attacks, previous 12-months

A.1.1: Baseline

Level-1 Model

Log [Number of mother-reported asthma-like $attacks_{ii}$] = $\pi_{0j} + e_{ij}$

Level-2 Model

$\pi_{0j} = \beta_{00} + \upsilon_{0j}$

A.1.2: Final model

Level-1 Model

Log [Number of mother-reported asthma-like $attacks_{ij}$] = $\pi_{0j} + \pi_{1j}$ *(t29 months) + π_{2j} *(t41 months) + π_{3j} *(concurrent poverty) + π_{4j}*(single parent family) + e_{ij}

Level-2 Model

$\pi_{0j} = \beta_{00} + \beta_{01}$*(birth rank=2nd) + B_{02}*(birth rank>=3rd) + β_{03}*(boy) + β_{04}*(mother's education= secondary) + β_{05}*(mother's education <secondary) + β_{06}*(mother =<19 years old) + β_{07}*(mother > 34 years old) + υ_{0j}

$\pi_{1j} = \beta_{10}$
$\pi_{2j} = \beta_{20}$
$\pi_{3j} = \beta_{30}$
$\pi_{4j} = \beta_{40}$

A.2-Child's health perceived by the mother as less than very good, previous 12-months

A.2.1: Baseline

Level-1 Model

Logit [$\pi_{\text{health < very good-ij}}$] = $\pi_{0j} + e_{ij}$

Level-2 Model

$\pi_{0j} = \beta_{00} + \upsilon_{0j}$

A.2.2: Final model

Level-1 Model

Logit [$\pi_{\text{health < very good-ij}}$] = $\pi_{0j} + \pi_{1j}$*(t17 months) + π_{2j}*(t29 months) + π_{3j}*(t41 months) + π_{4j}*(concurrent poverty) + π_{5j}*(uses daycare) + e_{ij}

Level-2 Model

$\pi_{0j} = \beta_{00} + \beta_{01}$*(birth rank=2nd) + β_{02}*(birth rank>=3rd) + β_{03}*(boy) + β_{04}*(mother immigrant) + β_{05}*(mother's education=secondary)+ β_{06}*(mother's education <secondary) + υ_{0j}

$\pi_{1j} = \beta_{10}$
$\pi_{2j} = \beta_{20}$
$\pi_{3j} = \beta_{30}$
$\pi_{4j} = \beta_{40;}$
$\pi_{5j} = \beta_{50}$

B-Poverty during previous round (Fit with HLM 6.0 software)

B.1-Number of mother-reported asthma-like attacks, previous 12-months

B.1.1: Baseline

Level-1 Model

Log [Number of mother-reported asthma-like attacks$_{ij}$] = $\pi_{0j} + e_{ij}$

Level-2 Model

$\pi_{0j} = \beta_{00} + \upsilon_{0j}$

B.1.2: Final model

Level-1 Model

Log [Number of attacks$_{ij}$] = $\pi_{0j} + \pi_{1j}$*(t29 months) + π_{2j}*(t41 months) + π_{3j}*(poverty$_{i-1}$) + π_{4j}*(concurrent poverty$_i$) + π_{5j}*(single parent family) + e_{ij}

Level-2 Model

$\pi_{0j} = \beta_{00} + \beta_{01}$*(birth rank=2nd) + β_{02}*(birth rank>=3rd) + β_{03}*(boy) + β_{04}*(mother's education =secondary) + β_{05}*(mother's education <secondary) + β_{06}*(mother =<19 years old) + β_{07}*(mother > 34 years old) + υ_{0j}

$\pi_{1j} = \beta_{10}$
$\pi_{2j} = \beta_{20}$
$\pi_{3j} = \beta_{30}$
$\pi_{4j} = \beta_{40}$
$\pi_{5j} = \beta_{50}$

B.2-Child's health perceived by the mother as less than very good, previous 12-months

B.2.1: Baseline

Level-1 Model

Logit [$\pi_{\text{health < very good-ij}}$] = $\pi_{0j} + e_{ij}$

Level-2 Model

$\pi_{0j} = \beta_{00} + \upsilon_{0j}$

B.2.2: Final model

Level-1 Model

Logit [$\pi_{\text{health <very good-ij}}$] = π_{0j} + π_{1j}*(t29 months) + π_{2j}*(t41 months) + π_{3j}*(poverty$_{i-1}$) + π_{4i}*(poverty$_i$) + π_{5i}*(uses daycare$_{ii}$) + e_{ii}

Level-2 Model

π_{0i} = β_{00} + B_{01}*(birth rank=2nd) + β_{02}*(birth rank>=3rd) + β_{03}*(boy) + β_{04}*(mother immigrant) + β_{05}*(mother's education=secondary)+ β_{06}*(mother's education <secondary) + υ_{0i}

$\pi_{1j} = \beta_{10}$
$\pi_{2j} = \beta_{20}$
$\pi_{3j} = \beta_{30}$
$\pi_{4j} = \beta_{40}$
$\pi_{5j} = \beta_{50}$

C- Cumulative poverty (Poverty classes with Mplus 5.2; Growth curves with HLM 6.0)

C.1-Number of mother-reported asthma-like attacks, previous 12-months

C.1.1: Baseline

Level-1 Model

Log [Number of mother-reported asthma-like attacks$_{ij}$] = π_{0j} + e_{ij}

Level-2 Model

$\pi_{0j} = \beta_{00} + \upsilon_{0j}$

C.1.2: Final model

Level-1 Model

Log [Number of mother-reported asthma-like attacks$_{ij}$] = π_{0j} + π_{1j} *(t29 months) + π_{2j} *(t41 months) + π_{3j} *(single parent family) + e_{ij}

Level-2 Model

π_{0j} = β_{00} + β_{01}*(birth rank=2nd) + B_{02}*(birth rank>=3rd) + β_{03}*(boy) + β_{04}*(mother's education= secondary) + β_{05}*(mother's education <secondary) + β_{06}*(mother =<19 years old) + β_{07}*(mother > 34 years old) + β_{08}*(transient poverty) + β_{09}*(chronic poverty) + υ_{0j}

π_{1j} = β_{10} + β_{11} *(transient poverty) + β_{12}*(chronic poverty)
π_{2j} = β_{20} + β_{21} *(transient poverty) + β_{22}*(chronic poverty)
$\pi_{3j} = \beta_{30}$

C.2-Child's health perceived by the mother as less than very good, previous 12-months

C.2.1: Baseline

Level-1 Model

Logit [$\pi_{\text{health < very good-ij}}$] = π_{0j} + e_{ij}

Level-2 Model

$\pi_{0j} = \beta_{00} + \upsilon_{0j}$

C.2.2: Final model

Level-1 Model

Logit [$\pi_{\text{health < very good-ij}}$] = π_{0j} + π_{1j}*(t17 months) + π_{2j}*(t29 months) + π_{3j}*(t41 months) + π_{4j}*(uses daycare) + e_{ij}

Level-2 Model

π_{0j} = β_{00} + β_{01}*(birth rank=2nd) + β_{02}*(birth rank>=3rd) + β_{03}*(boy) + β_{04}*(mother immigrant) + β_{05}*(mother's education=secondary)+ β_{06}*(mother's education <secondary) + β_{07}*(transient poverty) + β_{08}*(chronic poverty) + υ_{0j}

π_{1j} = β_{10}*(transient poverty) $_{+}$ β_{12}*(chronic poverty)
π_{2j} = β_{20}*(transient poverty) $_{+}$ β_{12}*(chronic poverty)
π_{3j} = β_{30}*(transient poverty) $_{+}$ β_{12}*(chronic poverty)
π_{4j} = β_{30}

Appendix 3: Fixed effects and level 2 variances from multilevel Poisson (number of mother-reported asthma-like attacks) and logistic regressions (maternal perception of health as less than very good)

Table A1: Fixed effects of concurrent poverty on number of mother-reported asthma-like attacks, multilevel Poisson regressions, Quebec (QLSCD, 1998-2001).

Fixed Effect	Coefficient	(Standard Error)	p-value
Intercept (t-17 months)	-5.375	(0.281)	0.000
**Level 1 predictors**			
Time			
t-29 months	-0.049	(0.032)	0.129
t-41 months	-0.465	(0.040)	0.000
Concurrent poverty			
Sufficient income (ref)			
Insufficient income	0.328	(0.122)	0.007
Type of family			
Two-parent family (ref)			
Single-parent family	0.463	(0.088)	0.000
**Level 2 predictors**			
Birth rank			
1st born (ref)			
2nd born	1.408	(0.289)	0.000
>= 3rd born	1.899	(0.396)	0.000
Gender			
Girl (ref)			
Boy	1.256	(0.267)	0.000
Mother's education			
College or University studies (ref)			
High school diploma	0.086	(0.340)	0.801
No high school diploma	-0.032	(0.378)	0.933
Mother's age			
<34 years (ref)			
>=35 years	-1.170	(0.412)	0.005

Table A2: Fixed effects of concurrent poverty on health perceived as less than very good, multilevel logistic regressions, Quebec (QLSCD, 1998-2001).

Fixed Effect	Coefficient	(Standard Error)	p-value
Intercept (ref: t-5months)	-4.555	(0.195)	0.000
**Level 1 predictors**			
Time			
t-17 months	0.438	(0.137)	0.002
t-29 months	0.276	(0.137)	0.044
t-41 months	0.297	(0.139)	0.032
Concurrent poverty			
Sufficient income (ref)			
Insufficient income	0.409	(0.142)	0.004
Use of daycare centre			
Does not use (ref)			
Uses	0.527	(0.146)	0.001
**Level 2 predictors**			
Birth rank			
1st born (ref)			
2nd born	0.787	(0.168)	0.000
>= 3rd born	0.576	(0.219)	0.009
Gender			
Girl (ref)			
Boy	0.276	(0.153)	0.071
Mother's immigrant status			
Non-immigrant or immigrant from European country (ref)			
Immigrant from non-European country	0.674	(0.271)	0.013
Mother's education			
College or University studies (ref)			
High school diploma	0.421	(0.191)	0.028
No high school diploma	0.634	(0.208)	0.003

Table B1: Fixed effects of previous round poverty on number of mother-reported asthma-like attacks, multilevel Poisson regressions, Quebec (QLSCD, 1998-2001).

Fixed Effect	Coefficient	(Standard Error)	P-value
Intercept (ref: t-17months)	-5.476	0.287	0.000
**Level 1 predictors**			
Time			
t-29 months	-0.033	0.033	0.313
t-41months	-0.457	0.040	0.000
Concurrent poverty			
Sufficient income (ref)			
Insufficient income	0.257	0.121	0.033
Previous period-poverty			
Sufficient income (ref)			
Insufficient income	0.223	0.085	0.009
Type of family			
Two-parent family (ref)			
Single-parent family	0.497	0.089	0.000
**Level 2 predictors**			
Birth rank			
1st born (ref)			
2nd born	1.454	0.294	0.000
>= 3rd born	1.941	0.399	0.000
Gender			
Girl (ref)			
Boy	1.303	0.271	0.000
Mother's education			
College or University studies (ref)			
High school diploma	0.080	0.342	0.814
No high school diploma	-0.166	0.386	0.667
Mother's age			
<34 years (ref)			
>=35 years	-1.162	0.415	0.006

Table B2: Fixed effects of previous round poverty on health perceived as less than very good, multilevel logistic regressions, Quebec (QLSCD, 1998-2001).

Fixed Effect	Coefficient	(Standard Error)	p-value
Intercept (ref: t-5months)	-4.330	0.221	0.000
**Level 1 predictors**			
Time			
t-29 months	-0.176	0.136	0.198
t-41 months	-0.162	0.130	0.212
Concurrent poverty			
Sufficient income (ref)			
Insufficient income	0.122	0.198	0.537
Previous period poverty			
Sufficient income (ref)			
Insufficient income	0.193	0.197	0.329
Use of daycare centre			
Does not use (ref)			
Uses	0.598	0.166	0.001
**Level 2 predictors**			
Birth rank			
1st born (ref)			
2nd born	0.758	0.213	0.001
>= 3rd born	0.555	0.285	0.051
Gender			
Girl			
Boy	0.289	0.197	0.141
Mother's immigrant status			
Non-immigrant or immigrant from European country (ref)			
Immigrant from non-European country	0.781	0.346	0.024
Mother's education			
College or University studies (ref)			
High school diploma	0.439	0.250	0.078
No high school diploma	0.731	0.268	0.007

Table C1: Fixed effects poverty class membership on number of mother-reported asthma-like attacks, multilevel Poisson regressions, Quebec (QLSCD, 1998-2001).

	**Latent classes of poverty experience**								
	Non-poor			Tansient poverty			Chronic poverty		
	Coefficient	(Standard Error)	p-value	Coefficient	(Standard Error)	p-value	Coefficient	(Standard Error)	p-value
Intercept (ref: t-17months)	-5.338	(0.302)	0.000	-0.034	(0.425)	0.937	0.892	(0.417)	0.032
**Level-1 predictors**									
Time									
t-29 months	-0.200	(0.052)	0.000	0.147	(0.161)	0.363	0.277	(0.070)	0.000
t-41months	-0.380	(0.058)	0.000	-0.070	(0.145)	0.630	-0.149	(0.082)	0.069
Type of family									
Two-parents family(ref)									
Single-parents family	0.407	(0.095)	0.000						
**Level-2 predictors**									
Birth rank									
1st born (ref)									
2nd born	1.475	(0.298)	0.000						
>= 3rd born	2.014	(0.420)	0.000						
Gender									
Girl (ref)									
Boy	1.232	(0.280)	0.000						
Mother's education									
University (ref)									
Secondary	-0.101	(0.364)	0.782						
No secondary diploma	-0.448	(0.451)	0.321						
Mother's age									
<34 years (ref)									
>=35 years	-1.145	(0.402)	0.005						

Table C2: Fixed effects poverty class membership on health perceived as less than very good, multilevel logistic regressions, Quebec (QLSCD, 1998-2001).

	**Latent classes of poverty experience**								
	Non-poor			Transient poverty			Chronic poverty		
	Coefficient	(Standard Error)	p-value	Coefficient	(Standard Error)	p-value	Coefficient	(Standard Error)	p-value
Intercept (ref:t-5months)	-4.655	(0.217)	0.000	0.427	(0.355)	0.229	1.284	(0.312)	0.000
**Level-1 predictors**									
Time									
t-17 months	0.704	(0.172)	0.000	-0.956	(0.441)	0.030	-0.735	(0.358)	0.040
t-29 months	0.392	(0.179)	0.028	-0.058	(0.366)	0.875	-0.532	(0.344)	0.121
t-41months	0.357	(0.179)	0.045	0.167	(0.367)	0.648	-0.584	(0.321)	0.068
Use of daycare									
Does not use (ref)									
Using	0.518	(0.151)	0.001						
**Level-2 predictors**									
Birth rank									
1st born (ref)									
2nd born	0.723	(0.173)	0.000						
>= 3rd born	0.578	(0.226)	0.011						
Gender									
Girl (ref)									
Boy	0.276	(0.156)	0.077						
Mother's immigration status									
Non-immigrant or immigrant from European country (ref)									
Immigrant from non-European country	0.600	(0.288)	0.037						
Mother's education									
University (ref)									
Secondary	0.398	(0.195)	0.041						
No secondary diploma	0.446	(0.220)	0.043						

D-Between children variability: Level 2 variances

	Level 2 (between children) Variances	
	Number of mother-reported asthma-like attacks	Health perceived < very good
**Concurrent poverty**		
From unit-specific model		
Null	3.190	1.430
Time variables	3.182	1.440
Time + poverty	3.163	1.386
Full model for concurrent poverty	3.071	1.353
From Laplace		
Null	6.739	3.057
Time variables	6.729	3.083
Time + concurrent poverty	6.687	2.924
Full model for concurrent poverty	6.532	2.804
**Previous period poverty**		
From unit-specific model		
Null	3.189	1.409
Time variables	3.171	1.410
Time + poverty	3.138	1.379
Full model for concurrent poverty	3.056	1.364
From Laplace		
Null	6.740	3.333
Time variables	6.636	3.337
Time + concurrent poverty	6.556	3.211
Full model for concurrent poverty	6.435	3.111
